# CRISPR-Cas9-driven genome editing in *Bacillus methanolicus* MGA3

**DOI:** 10.3389/fmicb.2025.1728984

**Published:** 2026-01-28

**Authors:** May L. K. Khider, Marta Irla, Marina Gil López, Anna Gispert, Thomas Konjetzko, Meliawati Meliawati, Jochen Schmid, Trygve Brautaset, Luciana Fernandes Brito

**Affiliations:** 1 Department of Biotechnology and Food Science, Norwegian University of Science and Technology (NTNU), Trondheim, Norway; 2 Department of Biological and Chemical Engineering, Aarhus University, Aarhus, Denmark; 3 Institute of Molecular Microbiology and Biotechnology, University of Münster, Münster, Germany

**Keywords:** *Bacillus methanolicus*, CRISPR-Cas9, genome editing, metabolic engineering, methanol

## Abstract

*Bacillus methanolicus* is a thermophilic methylotroph that uses methanol as a sustainable feedstock in biotechnological industry. Here, we developed the CRISPR-Cas9 genome editing tool for *B. methanolicus* MGA3. This one-plasmid system induces Cas9-mediated double-strand breaks and exploits native DNA repair: homologous recombination for scarless deletions and gene replacements, and error-prone end-joining repair for mutagenesis in the absence of a repair template. Consistent with end-joining activity, Cas9 cutting without a template resulted in reproducible small indels near the cleavage site. Using homology-directed repair, we deleted the *katA* and *ald* genes, confirmed the edits by genome sequencing, and demonstrated the expected loss of catalase and alanine dehydrogenase activities, respectively; both phenotypes were restored by complementation. For targeted gene insertion, the *katA* locus was replaced with *mcherry*, and successful integration was verified by PCR and increased mCherry fluorescence relative to the wild type. The system was further used to delete *spo0A* and replace it with *mrpf1*. The overall genome-editing efficiency exceeded 85%. This study demonstrates that the developed genome editing platform enables precise and efficient genetic modifications for metabolic engineering in *B. methanolicus*.

## 1 Introduction

There is an increased demand for alternative carbon sources that do not compete with food production and can replace sugar-based feedstocks in biotechnology. Next-generation feedstocks, such as the one-carbon (C_1_) substrates methane and methanol, are becoming increasingly popular due to their abundance and reduced environmental impact.

As a result of the growing interest in the use of methanol as an alternative feedstock in biotechnological production processes, native methanol-utilizing bacteria, such as *Bacillus methanolicus* and *Methylorubrum extorquens*, become candidates for industrial (Blombach et al., 2022; Klein et al., 2022). *B. methanolicus* MGA3 is a thermophilic bacterium, with optimal growth at 50 °C, making it suitable for industrial processes where cooling can be either an environmental, technical, or financial issue. The high cultivation temperature and methanol-based growth is also preventative of contamination, which is a cumbersome problem in large-scale operations. Its high growth rate and natural ability of *B. methanolicus* MGA3 and derived strains to overproduce the amino acids glutamate and lysine (up to 65 g/L in methanol fed-batch fermentations) are additional characteristics that make this bacterium a highly suitable host for bioprocesses (Brautaset et al., 2007; Heggeset et al., 2012).

This native methylotroph has been extensively studied for biotechnological applications and engineered using a range of genetic tools to produce value-added products (Ochsner et al., 2015; Irla et al., 2017; Drejer et al., 2020). The potential of *B. methanolicus* as a cell factory has been steadily advancing, and in recent years it has been engineered to produce various chemicals such as cadaverine and γ-aminobutyric acid (Irla et al., 2017; Nærdal et al., 2015). This progress was made possible by previously established genetic tools and scientific developments such as the use of a theta-replicating and rolling circle-replicating plasmid backbones for controlled gene expression, elucidation of genome sequence, and insights into the transcriptome, proteome, and metabolome (Müller et al., 2014; Irla et al., 2015, 2016, 2024; Delépine et al., 2020). These tools and insights can be used for further strain engineering and the stable over-expression of heterologous genes from either one or a set of two complementary plasmids. CRISPR interference (CRISPRi) was successfully applied to downregulate the expression of three genes related to sporulation, hydrogen peroxide (H_2_O_2_) detoxification, and mannitol catabolism in *B. methanolicus* (Schultenkämper et al., 2019). The CRISPRi-mediated gene downregulation is a robust and rapid method to study the function of targeted genes, however, with limited applicability for metabolic engineering as it often does not support genetic stability of production strains. For that reason, the method for genome modification has been a long-sought milestone for *B. methanolicus*. The difficulty of generating a genome modification tool for *B. methanolicus* may have been caused by the potential bottlenecks in its homologous recombination pathway and the low efficiency of transformation methods. Very recently, a deletion method based on homologous recombination was published for this organism where the genome modifications were enabled by the use of long fused homologous arms (2–6 kb) as a strategy for homologous recombination, combined with an optimized, highly efficient electroporation protocol (Li et al., 2025).

Recent works in progress have explored CRISPR applications in *B. methanolicus*, including reports of single-gene deletions, multiplex editing concepts, and integration strategies (Liu et al., 2025; Eikmeier, 2025). These early efforts reinforce growing interest in developing genetic tools for this organism. Our work builds on this momentum, aiming to expand genome deletion methods for *B. methanolicus* by employing the CRISPR-Cas9 technology, enabled by an efficient plasmid transformation via conjugation. Here, we report the use of the system developed by Rütering et al. (2017) for efficient CRISPR-Cas9-mediated genome editing in *B. methanolicus*. This system was first developed for *Paenibacillus polymyxa* and has since been successfully applied to diverse applications in this species (Meliawati et al., 2022a,b, 2024; Schilling et al., 2023; Ravagnan et al., 2024). We explored the native DNA repair mechanisms in *B. methanolicus*. In the absence of a repair template, an error-prone end-joining system ligates DNA ends, causing small insertions or deletions. More precise editing occurs via homology-directed repair (HDR) when a repair template is provided (Ran et al., 2013). In this study, we present a detailed demonstration of the functional CRISPR-Cas9-mediated genome modification system for two target genes: the catalase enzyme encoded by *katA*, and the gene *ald* that encodes alanine dehydrogenase (Ald). Furthermore, to validate the versatility of the developed tool, the master regulator gene *spo0A* was successfully deleted and replaced, underscoring its broad applicability for targeted genome modifications.

## 2 Materials and methods

### 2.1 Strains, media and growth conditions

Unless stated otherwise, all chemicals used in this study were purchased from Sigma-Aldrich. *Escherichis coli* DH5α was used as a general cloning host, and *E. coli* S17.1 λ*pir* for conjugal transfer of plasmids. *B. methanolicus* MGA3 was used as the expression host. All strains used in this study are listed in Table 1. *E. coli* strains were cultivated at 37 °C in either Lysogeny Broth (LB) or on LB agar plates, with supplementation of 50 μg/mL kanamycin or 25 μg/mL chloramphenicol when relevant. *B. methanolicus* was cultivated at 50 °C and 200 rpm in Super Optimal Broth (SOB; Difco) or SOB agar plates during strain development and otherwise cultivated in minimal media MVcM or MVcMY (0.25 g/L yeast extract) supplemented with 200 mM methanol. The MVcM medium contained, in 1 L of distilled water: 4.09 g K_2_HPO_4_, 1.49 g NaH_2_PO_4_*H_2_O, 2.11 g (NH_4_)_2_SO_4_; the pH was adjusted to 7.2 with NaOH before autoclaving. The MVcM medium was supplemented with 1 mL 1 M MgSO_4_*7H_2_O solution. Moreover, 1 mL trace element solution was added, containing in 1 L of distilled water: 5.56 g FeSO_4_*7H_2_0, 27.28 mg CuSO_4_*2H_2_O, 7.35 g CaCl_2_*2H_2_O, 40.50 mg CoCl_2_*6H_2_O, 9.90 g MnCl_2_*4H_2_O, 287.54 mg ZnSO_4_*7H_2_O, 48.40 mg Na_2_MoO4*2H_2_O, 30.92 mg H_3_BO_3_, and 80 mL HCl. One mL of a vitamin solution was added; it contained in 1 L of distilled water: biotin, thiamine hydrochloride, riboflavin, D-calcium pantothenate, pyridoxine hydrochloride, nicotinamide, 0.1 g each; p-aminobenzoic acid, 0.02 g; folic acid, vitamin B_12_ and lipoic acid, 0.01 g each (Schendel et al., 1990). Media for *B. methanolicus* cultivation was supplemented with antibiotics (kanamycin 25 μg/mL, chloramphenicol 5 μg/mL) when relevant. The strains were cultivated either in 250 mL Erlenmeyer flasks or in 24-well Duetz system plates, each well holding 10 mL (Haupka et al., 2021). The culture volumes corresponded to 10% of the total flask volume or 30% of the well volume. The optical density at 600 nm (OD_600_) of cell cultures was monitored with a WPA CO 8000 Biowave photometer. For testing H_2_O_2_ tolerance, *B. methanolicus* was cultivated in MVcM supplemented with increasing concentrations of H_2_O_2_ (0, 0.1, 0.5, 1, 2, or 3 mg/L). The cultivation was carried out in the BioLector Pro microbioreactor system (m2p-labs) at 50 °C, with a 48-well flower plate containing 1 mL culture and with a stirring speed of 1,200 rpm, humidity set to 85%, and a biomass gain set to 7. Cell biomass formation, based on scattered light, was monitored over 24 h.

**Table 1:** Bacterial strains and plasmids used in this study.

**Name**	**Characteristics**	**Reference**
**Strains**		
***Escherichia coli***		
DH5α	General cloning host. F-*thi*-1 *endA*1 *hsdR*17(r-,m-) *supE*44 _*lacU*169 (_80*lacZ*_M15) *recA*1 *gyrA*96 *relA*1	Stratagene
S17.1 λ*pir*	Chromosomally integrated RP4 plasmid. *(F^−^) RP4-2-Tc::Mu aphA::Tn7recAλpirlysogen*	(Simon et al., 1983)
***Bacillus methanolicus***		
WT	Wild-type *B. methanolicus* MGA3 strain	ATCC 53907
iCas	*B. methanolicus* harboring the piCas plasmid with deactivated *cas9* (*dcas9*) gene expression	(Schultenkämper et al., 2019)
i*ald*	*B. methanolicus* harboring the piCas-*ald* plasmid with *dcas9* gene expression and targeting the *ald* gene	This study
Δ*katA*	*B. methanolicus* with a scarless deletion of the catalase gene *katA*	This study
Δ*katA* + E	Δ*katA* strain harboring the plasmid pTH1mp	This study
Δ*katA* + *katA*	Δ*katA* strain harboring the plasmid pTH1mp-*katA*	This study
Δ*katA::mCherry*	*B. methanolicus* with a scarless deletion of *katA* replaced by an *mcherry* gene (Shaner et al., 2004). Constitutive gene expression under control of the standard *mdh* promoter from *B. methanolicus* (Irla et al., 2016). The P*mdh*-*mcherry* construct was amplified from the pTH1mp-*mcherry* plasmid	This study
*katA*_mut_	*B. methanolicus* with a mutation-derived inactivation of the catalase gene *katA*	This study
Δ*ald*	*B. methanolicus* with deletion a of the alanine dehydrogenase gene *ald*	This study
Δ*ald* + E	Δ*ald* strain harboring the plasmid pTH1mp	This study
Δ*ald* + *ald*	Δ*ald* strain harboring the plasmid pTH1mp-*ald*	This study
Δ*spo0A*	*B. methanolicus* with a scarless deletion of the Stage 0 sporulation protein A gene *spo0A*	This study
Δ*spo0A::mrfp1*	*B. methanolicus* with a scarless deletion of the Stage 0 sporulation protein A gene *spo0A*, replaced by an *mrfp1* gene (Campbell et al., 2002)	This study
**Plasmids**		
piCas	Kan^R^; pNW33NKan-based plasmid with *dcas9* gene and sgRNA expression driven by a *B. methanolicus mtlD* gene-derived mannitol inducible promoter	(Schultenkämper et al., 2019)
piCas-*ald*	piCas plasmid with sgRNA targeting the *ald* gene, with the sequence 5′-ggtgtgcagagagcaaaagt-3′ inserted at the *Ava*I x *Xba*I restriction site	This study
pCasPP	Kan^R^; pUB110-based plasmid, ori, *repU* gene, *cas9* under transcriptional control of the broad-host-range S-layer gene promoter *sgsE* from *Geobacillus stearothermophilus*, sgRNA region: constitutive *gapdh* promoter; BbsI flanked *lacZ* selection cassette	(Rütering et al., 2017)
pCasPP- *katA*	pCasPP with sgRNA targeting *B. methanolicus katA* gene (5′-aataagaaaaaacttactacaagc-3′); with fused homologous arms 1 kb upstream and 1 kb downstream *katA* inserted at the plasmid's *Spe*I site	This study
pCasPP-*ald*	pCasPP with sgRNA targeting *B. methanolicus ald* gene (5′-gattgtagacgtacgcggaa-3′); with fused homologous arms 1 kb upstream and 1 kb downstream *ald* inserted at the plasmid's *Spe*I site	This study
pCasPP-*spo0A*	pCasPP with sgRNA targeting *B. methanolicus spo0A* gene (5′-gtcatgcgatcgaagtagcg-3′); with fused homologous arms 1 kb upstream and 1 kb downstream *spo0A* inserted at the plasmid's *Spe*I site	This study
pCasPP-sg*katA*	pCasPP with sgRNA targeting *B. methanolicus katA* gene (5′-aataagaaaaaacttactacaagc-3′)	This study
pCasPP-*katA*::*mcherry*	pCasPP with sgRNA targeting *B. methanolicus katA* gene (5′- aataagaaaaaacttactacaagc-3′); with *mcherry* gene flanked by homologous arms 1 kb upstream and 1 kb downstream *katA* inserted at the plasmid's *Spe*I site	This study
pCasPP-*spo0A*::*mrfp1*	pCasPP with sgRNA targeting *B. methanolicus spo0A* gene (5′-gtcatgcgatcgaagtagcg−3′); with *mrfp1* gene flanked by homologous arms 1 kb upstream and 1 kb downstream *spo0A* inserted at the plasmid's *Spe*I site	This study
pTH1mp	CmR; derivative of pTH1mp-*lysC* for gene expression under control of the *mdh* promoter	(Irla et al., 2016)
pTH1mp-*mcherry*	pTH1mp-derivate with *mcherry* gene from *B. methanolicus* under control of *mdh* promoter	(Irla et al., 2016)
pTH1mp-*katA*	pTH1mp derivate with catalase gene from *B. methanolicus* under control of *mdh* promoter	This study
pTH1mp-*ald*	pTH1mp derivate with alanine dehydrogenase gene from *B. methanolicus* under control of *mdh* promoter	This study

### 2.2 Molecular cloning

Plasmids used in this study are listed in Table 1 and primers in Supplementary Table 1. Recombinant DNA work in *E. coli* was performed as described in Sambrook et al. (1989). Plasmid DNA was isolated using Promega's Wizard^®^ Plus SV Miniprep kit. Plasmid backbones and homologous flank regions were amplified with Takara Bio's CloneAmp^TM^ HiFi PCR premix. Qiaquick PCR Purification and Gel Extraction kits (Qiagen) were used for PCR purification and gel extraction. Fragments were joined by isothermal DNA assembly according to Gibson et al. (2009). Colony PCR was performed using either Phusion^®^ High-Fidelity DNA polymerase (New England Biolabs) or GoTaq^®^ polymerase (Promega). The plasmids pCasPP-*spo0A*, pCasPP-*katA* and pCasPP-*ald* were constructed as described by Rütering et al. (2017) by fusing 1 kb homologous arms flanking the targeted gene. The 1 kb length of homologous arms was chosen based on Rütering et al. (2017). Twenty four bp long sgRNA were inserted into the sgRNA region of pCasPP plasmid, specifically at the *Bbs*I restriction site, by PCR overlap extension, and the plasmids were re-circularized using Gibson Assembly (Gibson et al., 2009). For the pCasPP-*katA::mcherry* and pCasPP-*spo0A::mrfp1* constructs, the *mcherry* and *mrfp1* genes were cloned between the homologous flanks via overlap extension PCR, and the same sgRNAs as for *katA* and *spo0A* deletions were used for gene replacement. CRISPRi plasmids were constructed by inserting 24 bp sgRNA in the XbaI/AvaI restriction sites of the piCas plasmid, following the method described by Schultenkämper et al. (2019). The genome sequence of *B. methanolicus* (GenBank accession numbers CP007739, CP007741, and CP007740) was used to identify 24-nucleotide target sequences on the non-translated strand using the CRISPR Guide RNA Design Tool. We selected sgRNAs with an *in silico* off-target score of 50, indicating high predicted specificity (Benchling, https://www.benchling.com/crispr).

*B. methanolicus* strains were transformed by utilizing conjugal transfer of plasmids from *E. coli* S17.1 λ*pir*, described by Irla et al. (2025). Recipient and donor cultures of *B. methanolicus* and *E. coli* S17.1 λ*pir* were cultivated overnight in 25 mL of SOB and LB media, respectively. Fourty mL 1% cultures of the donor and recipient cells were cultivated the next day for 4 hours before mixing them in two dilutions: 9 mL of *B. methanolicus* with 3 mL of *E. coli* S17.1 λ*pir*, and 900 μL of *B. methanolicus* with 300 μL *E. coli* S17.1 λ*pir*. The mixes were centrifuged at 8,000 rpm for 5 min and resuspended gently in 100–200 μL of leftover supernatant. The cell mixtures were then transferred without spreading to a non-selective SOB agar plate and incubated overnight at 40 °C. The mixed cell colonies were then collected and resuspended in 200 μL of prewarmed SOB media and plated on selective SOB agar at 50 °C. Transconjugant colonies (*n* = 20) were replated, and leftover cell material was boiled in water for 10 min at 98 °C and vortexed. The cells were then centrifuged at 13,000 rpm for 5 min in a Mini Spin centrifuge (Eppendorf). The supernatant was then diluted ten times before being used as a template in PCR for screening deletion clones. If still present, the pCaspp-based plasmids were curated through 4 successive cultivations in antibiotic-free SOB liquid medium. To verify plasmid curing, deletion clones were plated on selective SOB plates, and the absence of growth confirmed successful plasmid loss. Furthermore, a Monarch Spin gDNA Extraction kit (New England Biolabs) was used to extract genomic DNA of positive colonies, according to the manufacturer's recommendations. The correctness of all cloning and gene deletions was confirmed via Sanger sequencing by a third-party (Eurofins Genomics).

### 2.3 Next-generation sequencing and variant calling

For whole genome sequencing, one representative of each deletion clone was cultured overnight in SOB medium and genomic DNA was isolated using the Monarch Spin gDNA Extraction kit. Whole genomes were sequenced by a third-party (Eurofins Genomics). Illumina sequencing was conducted by constructing two paired-end libraries with average insertion lengths of 500 bp and 2,000 bp. Sequences were generated with an Illumina GA IIx (Illumina Inc., San Diego, CA, USA). Raw data was processed in four steps, including removing reads with 5 bp of ambiguous bases, removing reads with 20 bp of low quality (≤Q20) bases, removing adapter contamination, and removing duplicated reads. Finally, 100× libraries were obtained with clean paired-end read data.

Sequencing reads were mapped to the reference genome sequence, composed of the chromosome and the two native plasmids pBM19 and pBM69 (GenBank accession numbers CP007739, CP007741, and CP007740, respectively). Before the alignment, the reads underwent trimming with a minimum length of 36 bp using the Trimmomatic v0.33 tool (Bolger et al., 2014). The trimmed reads were then aligned to the reference sequences using the short-read alignment software Bowtie 2 (Langmead et al., 2009). Mapped reads were visualized employing the ReadXplorer v2.2.2 tool (Hilker et al., 2016). Variant calling was conducted using the Bcftools mpileup pipeline, with quality score cut-off set to 50 (Li et al., 2009).

### 2.4 Enzyme activity assays

*B. methanolicus* strains cultured in MVcMY media were harvested at OD_600_ 1–2 by centrifugation at 8,000 rpm and 4 °C for 10 min in an Eppendorf 5430 R centrifuge. Cell pellets were washed twice and resuspended in 50 mL Tris-HCl pH 7.5 before storing at -80 °C. The cells were then thawed on ice and transferred to 2 mL screw-cap tubes containing 200 μL 0.1 mm glass beads (Carl Roth) and disrupted in three rounds using a Retsch MM400 bead beater at 30 ms for 1 min and resting on ice for 5 min. The crude extract was isolated after centrifugation at 17,000 rpm for 1 h at 4 °C in an Eppendorf 5424 R centrifuge. Protein concentrations were determined with Bradford assay protocol (Bio-Rad). To determine catalase activity, the crude extracts underwent catalase assay using the Catalase Colorimetric Activity Kit (Thermo Fisher), according to the manufacturer's protocol. Furthermore, H_2_O_2_ drop assay was performed by adding 3 μL of 3 % (v/v) H_2_O_2_ to 150 μL of a cell culture (OD_600_ ≈ 2) in a Thermo Scientific™ Nunc MicroWell 96-Well optical-bottom microtiter plates. The production of oxygen could be detected by the formation of bubbles over the mixture (Schultenkämper et al., 2019).

Ald assay reactions measuring the reductive amination of pyruvate contained final concentrations of 50 mM Tris-HCl, pH 7.5, 5 mM pyruvate, 0.5 mM NADH, 200 mM NH_4_Cl, and 50 μL of crude extract in a total volume of 200 μL. Ald assay reactions measuring the oxidative deamination of alanine contained 50 mM Tris-HCl pH 7.5, 5 mM alanine, 0.5 mM NAD^+^, and 50 μL crude extract. The reactions were monitored at 340 nm for 6 min under a constant temperature of 40–42 °C. Calculations were performed based on a molar extinction coefficient of 3.990 L/mmol/cm for NADH. Both Ald assays were conducted using a TECAN-Infinite M200-Microplate reader and Thermo Scientific™ Nunc MicroWell 96-Well optical-bottom plates.

### 2.5 Analysis of extracellular amino acids in *B. methanolicus* supernatants

Supernatants from *B. methanolicus* strains cultured in MVcM media were collected (1 mL) by centrifugation at 8,000 rpm and room temperature for 10 min in an Eppendorf 5430 R centrifuge and stored at -20 °C until use. The supernatants were analyzed using high pressure liquid chromatography (HPLC) samples were derivatized using a Waters Alliance e2695 Separations Module. The samples were derivatized using FMOC-Cl (fluorenylmethyloxycarbonyl chloride), according to Melucci et al. (1999). Amino acids were separated in a Symmetry C18 column (100 Å, 3.5 μm, 4.6 mm × 75 mm, Waters) carried by mobile phase A, 50 mM Na-acetate, pH 4.2, and B, acetonitrile. The mobile phase flow rate was set to 1.3 mL/min and the gradient conditions were as follows: 5 min- 62% A and 38% B, 12 min- 43% A and 57% B, 14 min-24% A and 76% B, 15 min-43% A and 57% B, and 18 min- 62% A and 38% B. The detection was performed with a Waters 2475 HPLC Multi Fluorescence Detector (Waters), with excitation at 265 nm and emission at 315 nm (Brito et al., 2019).

## 3 Results

### 3.1 Development of the CRISPR-Cas9-mediated genome modification system and its application to delete the chromosomal *ald* and *katA* genes

The CRISPR-Cas9 mediated genome modification tool developed in this study is based on the pUB110-based plasmid pCasPP, carrying a *Streptococcus pyogenes-*derived c*as9* gene under the transcriptional control of the broad-host-range surface layer protein gene (*sgsE*) promoter from *Geobacillus stearothermophilus*, and an sgRNA region under a constitutive *gapdh* promoter derived from *Streptomyces griseus*. It carries a neomycin/kanamycin resistance gene, a *repU* gene responsible for replication in Bacillales, *Bbs*I flanked *lacZ* selection cassette, and origin of transfer (*oriT*) required for conjugation (Shao et al., 2013; Cobb et al., 2015). The plasmid contains a *Spe*I site which is used for insertion of homologous arms necessary for HDR (Rütering et al., 2017). The Cas9-carrying plasmids can be transferred to *B. methanolicus* cells via electroporation or conjugation with *E. coli* S17.1 λ*pir* through a co-cultivation step carried out at 40 °C. The Cas9 activity threshold lies below the optimal growth temperature of *B. methanolicus* at 50 °C (Wiktor et al., 2016; Mougiakos et al., 2017a; Schmidt et al., 2019). Therefore, lowering the temperature to 40 °C during conjugation with *E. coli* not only allowed co-cultivation on the two strains but also activity of Cas9 protein. The subsequent raising of the temperature to 50 °C led to elimination of the *E. coli* cells and deactivation of Cas9 protein. The use of a more efficient plasmid delivery method reduces the screening burden. Therefore, this conjugation method yields approximately 600 colonies on plate, compared to ~20 colonies achieved using the classic electroporation technique (Irla et al., 2025). To validate the functionality of the CRISPR-Cas9 based pCasPP system for this thermophilic bacterium, we targeted two distinct genes located at different loci in the *B. methanolicus* genome: the *katA* gene (BMMGA3_04865; WP_004433703.1), which encodes a catalase, and the *ald* gene (BMMGA3_13155; WP_003347426.1), which encodes an alanine dehydrogenase.

**Figure 1:**
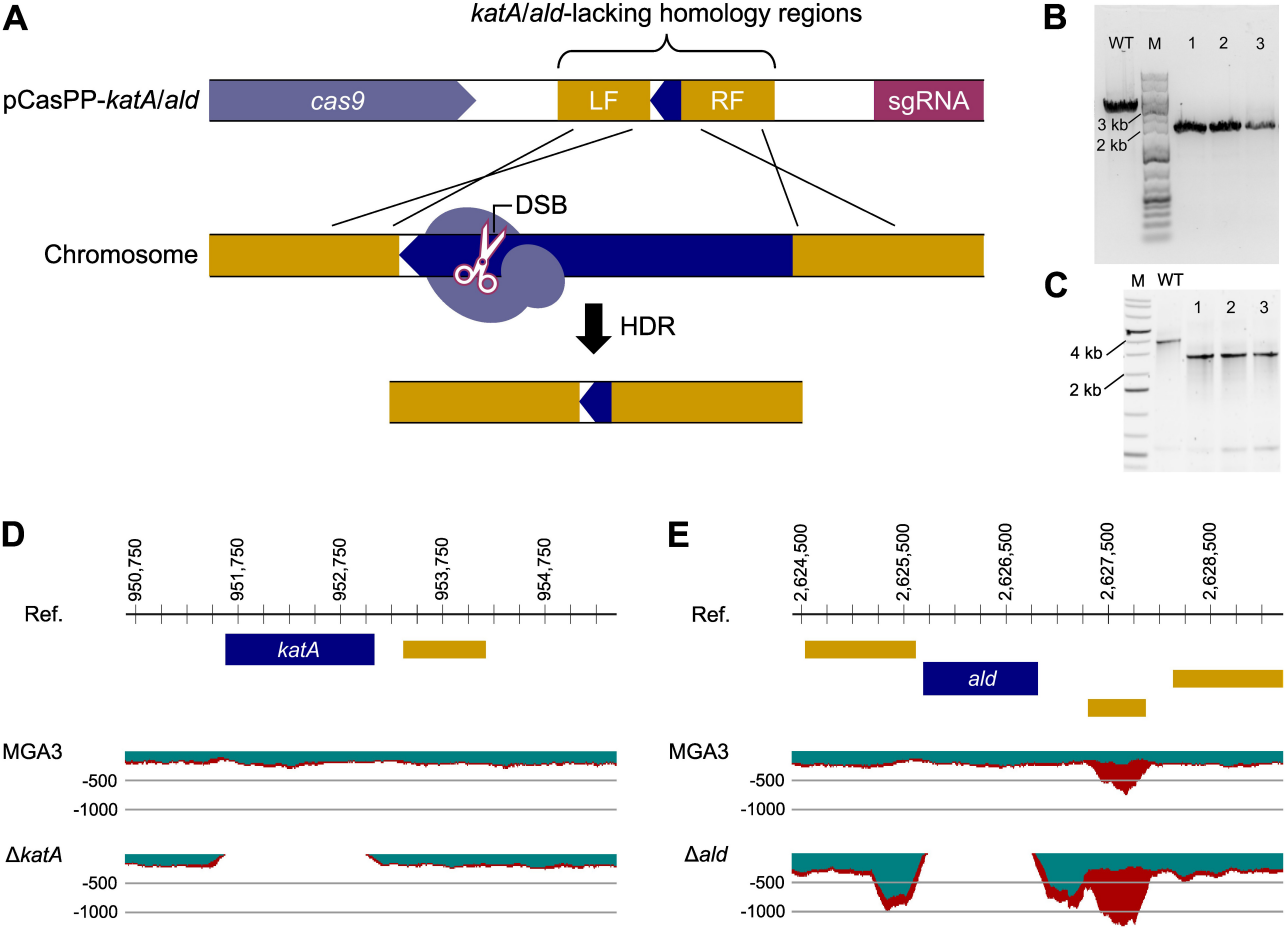
CRISPR-Cas9-mediated deletion method for *B. methanolicus* MGA3. **(A)** The pCasPP plasmid introduced into *B. methanolicus* carries the Cas9 coding sequence, a sgRNA designed to target a specific DNA sequence, and a template DNA sequence flanked by homologous regions corresponding to the target DNA. The CRISPR-associated enzyme Cas9 breaks down the target DNA in the chromosome to create a double-strand break DSB. DSB repair includes the homology directed repair (HDR) pathway that uses DNA template sequences to create precise short or long insertions from the plasmid DNA. The CRISPR-Cas9-mediated deletion was confirmed by means of screening comprising amplification of genome regions flanking the template's homologous arms: **(B)** WT ~3.5 kb DNA fragment opposed to ~2.5 kb DNA fragments from *katA* deletion clones; **(C)** WT ~3.5 DNA fragment opposed to ~2.5 kb DNA fragments from *ald* deletion clones. Genome deletions were further confirmed employing complete genome sequencing (**D** and **E**).

In a previous study, the role of *katA* was analyzed using the CRISPRi approach. Targeting *katA* with CRISPRi led to a decrease in catalase activity by about 75% (Schultenkämper et al., 2019). Here, we leveraged the CRISPRi system to test the phenotypical response of targeting the *ald* gene (Supplementary Figure 1A). Repression of *ald* resulted in a decrease by 12% in alanine production under small-scale Duetz plate conditions (Supplementary Figure 1B). CRISPRi results provided preliminary confirmation of the target activities, supporting their potential for further deletion experiments. For gene deletion, we designed sgRNA sequences targeting the same *katA* and *ald* genes along with homologous regions of around 1,000 bp for each flank to obtain scarless deletions in *B. methanolicus* (Figure 1A) (Rütering et al., 2017). The plasmids named pCasPP-*katA* and pCasPP-*ald* were constructed (Table 1) and used to transform *B. methanolicus* by conjugation.

For the confirmation of gene deletions in *B. methanolicus*, PCR amplification of genomic DNA was performed using primers flanking the homology regions incorporated in the deletion plasmid. The resulting amplicons as visualized by gel electrophoresis confirmed a reduction in fragment size for both the Δ*katA* and Δ*ald* deletions compared to the wild-type (WT) control, as shown in Figures 1B, C. The deletion efficiency was approximately 87%, with 2–3 negative clones routinely observed among 20 randomly picked colonies, which represents a total of 5.22 × 10^−6^ recombinants per cell (data not shown). To further confirm the gene deletions, the genomic DNA from the deletion strains Δ*katA* and Δ*ald*, and the WT control strain was sequenced and the reads mapped to the reference genome. While reads derived from the WT strain mapped continuously across the *katA* and *ald* regions, the deletion strains exhibited clear unmapped gaps in these regions, indicating the successful CRISPR-mediated deletion of the target genes (Figures 1D, E).

### 3.2 Deletion of *katA* resulted in loss of catalase activity and depleted H_2_O_2_ tolerance

**Figure 2:**
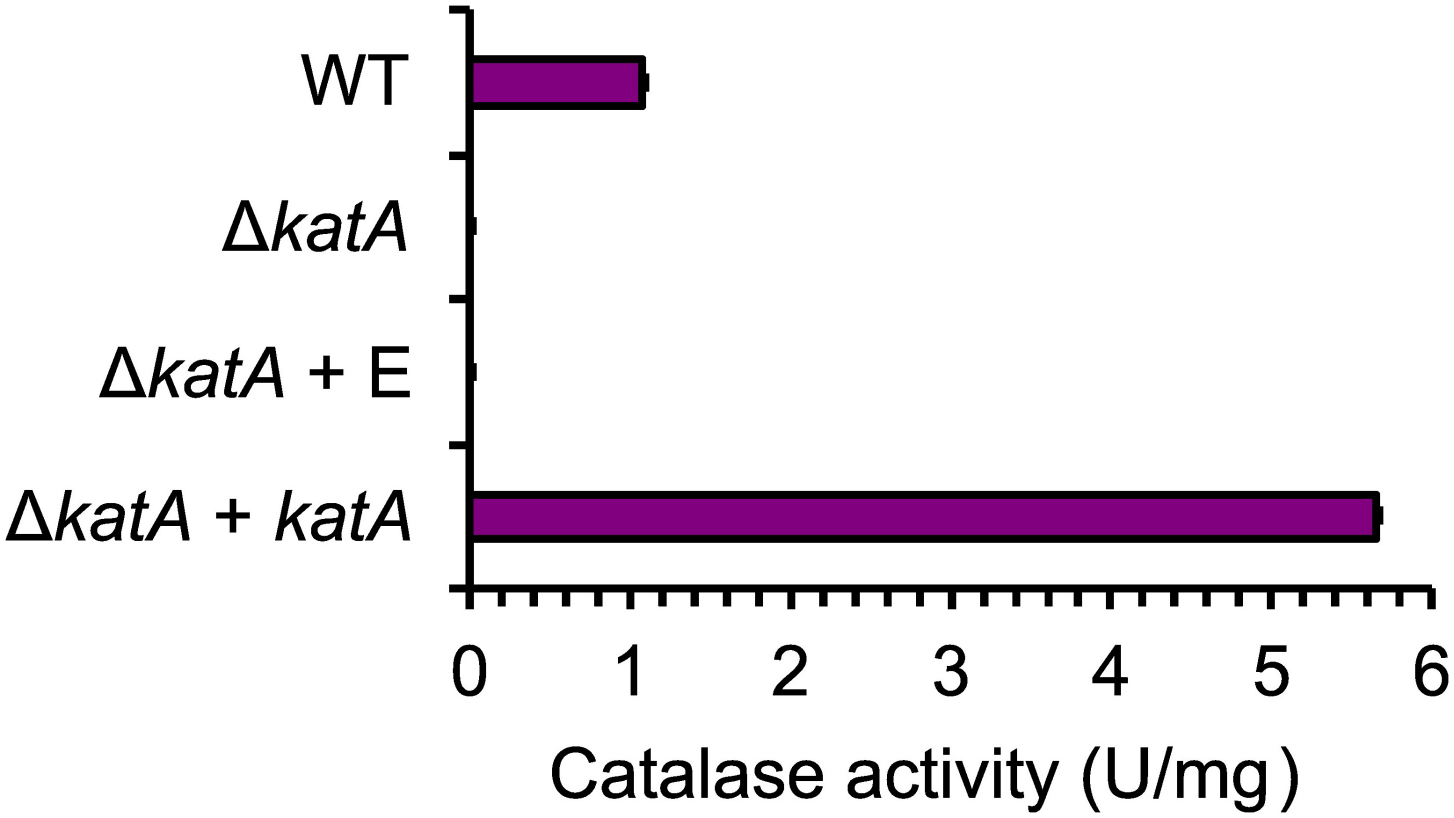
Catalase specific activity measured in *B. methanolicus* strains WT, Δ*katA*, Δ*katA* + E and Δ*katA* + *katA*. Δ*katA* + E refers to the Δ*katA* strain transformed with the empty vector pTH1mp, while Δ*katA* + *katA* refers to the Δ*katA* strain transformed with the complementation plasmid pTH1mp-*katA*. One unit **(U)** is defined as μM H_2_O_2_ decomposed per minute. Bars represent the mean specific catalase activity (U/mg) of technical triplicates, with error bars indicating the standard error.

In this study, we deleted the catalase-encoding gene *katA* and assessed its role in H_2_O_2_ detoxification in *B. methanolicus*. The specific catalase activity was measured in crude extracts from the WT strain and the catalase deletion strain Δ*katA*. Additionally, catalase activity was verified in the complementation strain Δ*katA* + *katA* and its empty vector counterpart, Δ*katA* + E (Table 1). The catalase activity in the WT strain was ~1.2 U/mg. In contrast, the *katA* deletion resulted in a loss of any detectable catalase activity, as observed in both Δ*katA* and Δ*katA* + E strains. As expected, the complementation strain exhibited significantly elevated catalase activity (~5.8 U/mg), driven by plasmid-based gene expression (Figure 2).

**Figure 3:**
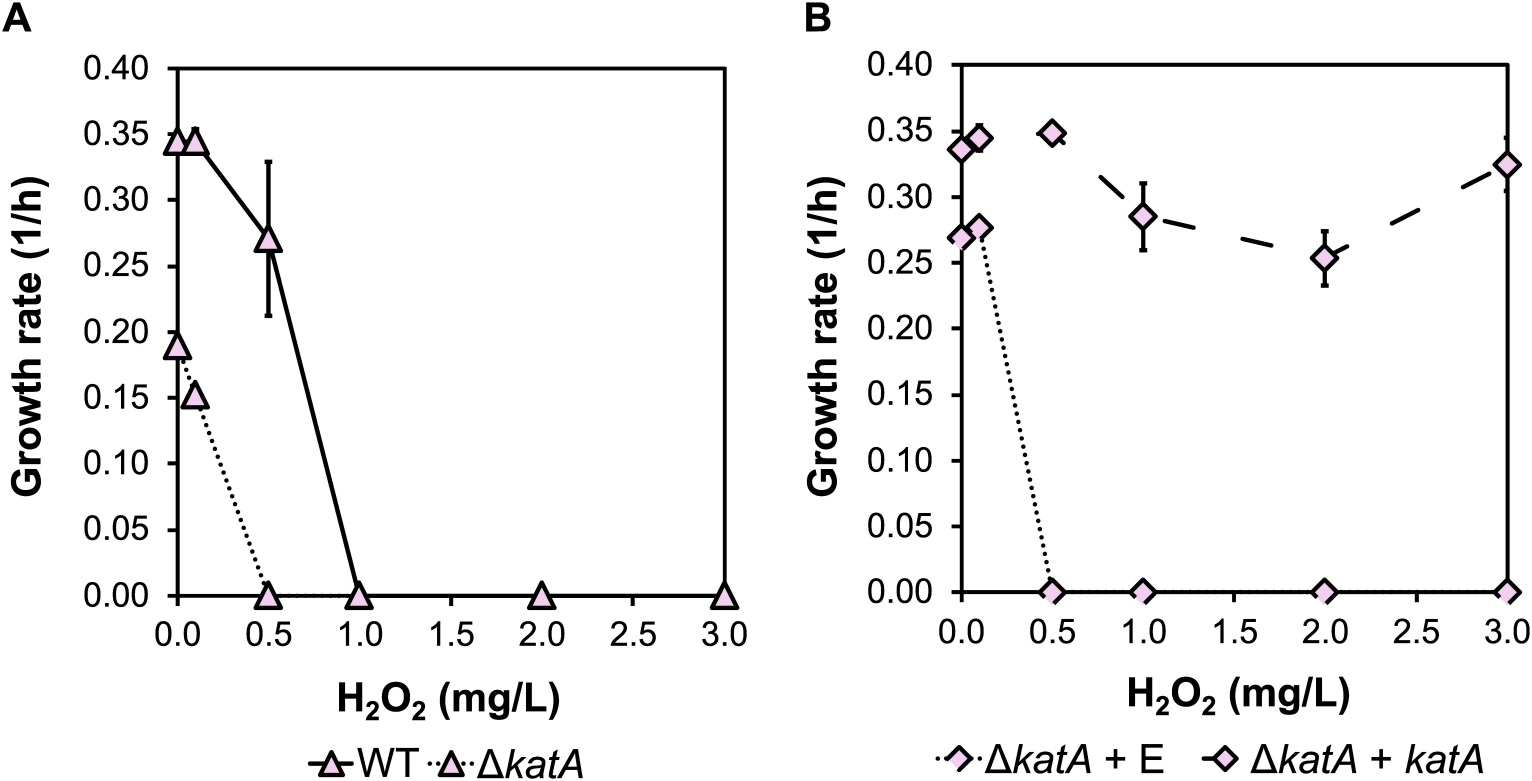
Growth rates of *B. methanolicus* strains under increasing concentrations of H_2_O_2_. The *B. methanolicus* strains WT and Δ*katA* **(A)**, and Δ*katA* + E and Δ*katA* + *katA* **(B)** were cultivated in the Biolector Pro system with flower plates containing methanol minimal media supplemented with gradually increasing concentrations of H_2_O_2_ (0, 0.1, 0.5, 1, 2 or 3 mg/L). Δ*katA* + E refers to the Δ*katA* strain transformed with the empty vector pTH1mp, while Δ*katA* + *katA* refers to the Δ*katA* strain transformed with the complementation plasmid pTH1mp-*katA*. Means of technical triplicates are represented by triangles or diamonds, with error bars indicating the standard error.

We further evaluated the impact of *katA* deletion under oxidative stress by assessing cell fitness in response to increasing concentrations of H_2_O_2_ supplemented into the growth media. The strains were cultivated in a microbioreactor (Biolector Pro system plates). The growth rates of *B. methanolicus* strains (WT, Δ*katA*, Δ*katA* + E, and Δ*katA* + *katA*) were measured under H_2_O_2_ concentrations ranging from 0 to 3 mg/L, which allowed us to investigate the role of catalase in H_2_O_2_ detoxification. Our results indicate that WT *B. methanolicus* MGA3 naturally tolerates up to 0.5 mg/L H_2_O_2_, as the WT strain exhibited sustained growth under these conditions. However, growth of the WT strain was impaired at higher concentrations of H_2_O_2_ (Figure 3A). The Δ*katA* deletion strain showed an ~21% reduction in growth rate at 0.1 mg/L H_2_O_2_ compared to the no-supplement control. Moreover, approximately 42% growth reduction occurred for the Δ*katA* strain in comparison to the WT even without ant H_2_O_2_ supplementation, indicating the importance of catalase for the cell fitness (Figure 3A). Growth in the Δ*katA* strain was completely halted starting from the supplementation of 0.5 mg/L mM H_2_O_2_. In the Δ*katA* + E strain, which carries the empty vector plasmid, a similar growth pattern was observed, with growth maintained only up to 0.1 mg/L H_2_O_2_. We suggest that a minor residual oxidative stress tolerance in the Δ*katA* strain may result from alternative paralogs. A BLAST search identified two thiol-dependent peroxidases (BMMGA3_03510 and BMMGA3_05260) that may play a role in oxidative stress defense in *B. methanolicus* MGA3. Additionally, four genes annotated with the Gene Ontology term GO:0004601, indicating peroxidase activity, were found. However, the genome of *B. methanolicus* contains a single gene annotated as a catalase. In contrast, when the *katA* gene was complemented in the Δ*katA* + *katA* strain, the growth fitness was restored, with growth rates comparable across all H_2_O_2_ concentrations tested (Figure 3B). Thus, it is confirmed that the deletion of *katA* was responsible for the low H_2_O_2_ tolerance phenotype.

### 3.3 Deletion of *ald* resulted in decreased—but not abolished—alanine dehydrogenase activity

In *B. subtilis*, alanine dehydrogenase catalyzes the reversible reductive amination of pyruvate to alanine (Supplementary Figure 2A). The *B. methanolicus* strains WT, Δ*ald*, Δ*ald* + E, and Δ*ald* + *ald* (Table 1) were tested for Ald activity. Enzyme activity was measured in crude cell extracts to evaluate whether deletion of the *ald* gene in *B. methanolicus* impairs the oxidative deamination of alanine and to examine potential directional preferences. Hence, the consumption or accumulation of the reaction cofactor NADH was monitored. The specific enzyme activities are presented in Table 2. The deletion strains Δ*ald* and Δ*ald* + E displayed comparable levels of Ald activity for the reductive amination of pyruvate (2.8 ± 0.5 mU/mg and 4.3 ± 1.8 mU/mg), which was five-fold lower compared to the control strain WT. Whereas the over-production of Ald in the complemented deletion strain (Δ*ald* + *ald*) lead to a more than 200-fold increase in activity at 4,709 ± 200 mU/mg. Interestingly, in the oxidative deamination of alanine, only Δ*ald* + *ald* displayed activity, which was substantially reduced in comparison to the activity displayed in the same strain for the reverse reaction (Table 2).

**Table 2:** Specific enzyme activity in mU/mg of Ald in the reductive amination of pyruvate and the oxidative deamination of alanine, where one U (unit) is the amount of enzyme catalyzing the oxidation of 1 mmol NADH/minute or reduction of 1 mmol NAD^+^/minute, respectively.

**Strain**	**Pyruvate amination (mU/mg protein)**	**Alanine deamination (mU/mg protein)**
WT	17.3 ± 4.0	0.00 ± 0.00
Δ*ald*	2.8 ± 0.5	0.00 ± 0.00
Δ*ald +* E	4.3 ± 1.8	0.00 ± 0.00
Δ*ald +ald*	4,709 ± 200	0.14 ± 0.01

Δ*ald* + E refers to the Δ*ald* strain transformed with the empty vector pTH1mp, while Δ*ald* + *ald* refers to the Δ*ald* strain transformed with the complementation plasmid pTH1mp-*ald*. The results show the average of technical triplicates ± standard deviation.

Furthermore, to assess the effects of *ald* deletion in *B. methanolicus*, the accumulation of alanine was measured in the strains WT, Δ*ald*, Δ*ald* + E, Δ*ald* + *ald* grown in minimal media in 10-well Duetz plates system. The results from CRISPRi-mediated repression of the *ald* gene led to significant changes in alanine titer in *B. methanolicus* strain i*ald*, with a reduction in alanine production in comparison to the non-targeting control (Supplementary Figure 1B). However, the deletion of *ald* did not replicate the effects observed with gene repression. The WT strain secreted approximately 168 mg/L of alanine, while the Δ*ald* and Δ*ald* + E strains produced around 204 mg/L and 211 mg/L of alanine, respectively. The complementation strain produced about 142 mg/L of alanine. Although slight trends were observed between the strains, no significant differences (*p* < 0.01) were detected in the Scott-Knott test (Supplementary Figure 2B). These findings suggest that while *ald* deletion may slightly affect alanine production, its impact on biosynthesis is not substantial enough to reach statistical significance under the conditions tested. Further studies may be needed to fully elucidate the role of *ald* in alanine metabolism.

### 3.4 Evaluating secondary mutations upon genome editing in *B. methanolicus* via variant calling

To analyse putative genetic variants derived from the CRISPR-Cas9 system, the genome sequences of the deletion strains Δ*katA* and Δ*ald*, as well as the WT were used to conduct the variant calling analysis in comparison to the *B. methanolicus* MGA3 reference genome sequence (GenBank accession numbers CP007739, CP007741, and CP007740 for chromosome and plasmids pBM19 and pBM69, respectively) (Irla et al., 2014). Variants that were shared between the WT and deletion strains were not included in Table 3, as they are inherent to the WT genome (Supplementary Table 2). Consequently, Table 3 presents only the deletion-associated mutations. Here, we identified 13 variants in the Δ*katA* strain and 14 variants in the Δ*ald* strain (Table 3). These variants represent deviations from the reference genome that do not appear in the WT sequence. These findings suggest that both deletions resulted in similar genetic plasticity, regardless the distinct locations and putative functions of the targeted genes. Notably, both deletion strains harbored variants in genes encoding enzymes involved in insertional mutagenesis and DNA repair processes. Alterations in DNA repair mechanisms have been shown to increase mutation rates in *B. subtilis* (Dervyn et al., 2023). Here, the Δ*katA* strain exhibited indel mutations in genes encoding SAM-dependent methyltransferases and DNA gyrase *gyrA*. The *ald* deletion strain presented a mutation in the gene encoding methionine-tRNA ligase *metG* (Table 3).

**Table 3:** Nucleotide variants determined by whole-genome sequencing.

**Reference**	**POS**	**REF**	**ALT**	**QS**	**EF**	**Feature**	**Product**
Δ***katA***							
Chromosome	339,328	C	CT	52	INDEL	*dagK*	Diacylglycerol kinase
	775,528	A	ATCT	52	INDEL	*yheD*	Endospore coat-associated protein YheD
	851,796	T	TC	70	INDEL	*asnO*	Asparagine synthetase [glutamine-hydrolyzing] 3
	933,664	AT	ATCTT	70	INDEL	BMMGA3_04740	Hypothetical protein
	1,027,104	T	C	62	NR	BMMGA3_05240	Hypothetical protein
	1,441,600	GAT	GATCTAT	70	INDEL	BMMGA3_07270	SAM-dependent methyltransferases family protein VrlL
	1,844,458	T	A	54	NR	BMMGA3_09230	Hypothetical protein
	2,890,416	AC	ACTC	75	INDEL	BMMGA3_14615	Mg^2+^ transporter protein CorA family protein
	2,896,912	TT	TTT	52	INDEL	*gpmI*	2,3-Bisphosphoglycerate-independent phosphoglycerate mutase
	3,159,842	A	ACT	75	INDEL	*gapN*	NADP-dependent glyceraldehyde-3-phosphate dehydrogenase
	3,228,083	GG	G	56	INDEL	*mmgA*	Acetyl-CoA acetyltransferase
pBM69	8,889	AT	ATCTT	70	INDEL	*gyrA*	DNA gyrase subunit A
	61,062	C	CT	70	INDEL	BMMGA3_00310	Hypothetical protein
*Δ**ald***							
Chromosome	46,330	T	A	102	NR	*metG*	Methionine-tRNA ligase
	188,692	c	cATCT	67	INDEL	*yisY*	AB hydrolase superfamily protein YisY
	411,168	T	G	68	NR	BMMGA3_02115	Hypothetical protein
	1,255,033	G	A	62	NR	*dapG*	Aspartokinase 1
	1,255,034	C	T	62	NR	*dapG*	Aspartokinase 1
	1,255,036	A	T	57	NR	*dapG*	Aspartokinase 1
	1,301,716	T	C	55	NR	BMMGA3_06590	TIGR00027 family putative methyltransferase
	1,465,073	A	C	62	NR	*thiM1*	Hydroxyethylthiazole kinase
	2,171,787	A	T	99	NR	BMMGA3_10940	Hypothetical protein
	2,508,869	g	gATC	65	INDEL	BMMGA3_12640	Hypothetical protein
	2,632,543	C	T	75	NR	BMMGA3_13185	Hypothetical protein
	2,867,122	ccg	cCGATCTcg	70	INDEL	BMMGA3_14500	Sortase family protein
	2,964,441	t	tCCGA	58	INDEL	*fliD*	Flagellar capping protein
	3,208,085	gc	gCAGATc	54	INDEL	*prfA*	Peptide chain release factor 1

Non-silent variants occurring in the *B. methanolicus* strains Δ*katA* and Δ*ald* in comparison to the WT strain. POS, position of single-nucleotide polymorphism (SNP) in the genome; REF, the sequence in the reference genome; ALT, sequence in the mutant strain genome; QS, quality score; EF, SNP effect; INDEL, small insertions and deletion; NR, non-redundant SNP.

Regarding the mutations potentially associated with the loss-of-function effects of gene deletions, the absence of catalase activity in the *katA* deletion strain resulted in mutations in enzymes and metabolic pathways that rely on water as a cofactor. The accumulation of H_2_O_2_ could interfere with these pathways, as it may modify enzyme active sites or other critical components, thereby disrupting their function. Hence, such mutated genes identified in the *katA* deletion strain included asparagine synthetase [glutamine-hydrolyzing] 3 (*asnO*), NADP-dependent glyceraldehyde-3-phosphate dehydrogenase (*gapN*), and 2,3-bisphosphoglycerate-independent phosphoglycerate mutase (Table 3). Surprisingly, the *ald* deletion strain displayed mutations in the *dapG* gene, which encodes aspartokinase 1, the enzyme catalyzing the phosphorylation of aspartate in lysine biosynthesis. The synthesis of lysine precursor, oxaloacetate, through carboxylation of pyruvate in an anaplerotic node is a competitive pathway to the alanine dehydrogenase reaction (Nærdal et al., 2017). However, the biological relationship between the *ald* deletion and the mutation in *dapG* remains unclear and warrants further investigation.

### 3.5 The CRISPR-Cas9-mediated genome modification system can be expanded for gene deactivation and gene insertions

**Figure 4:**
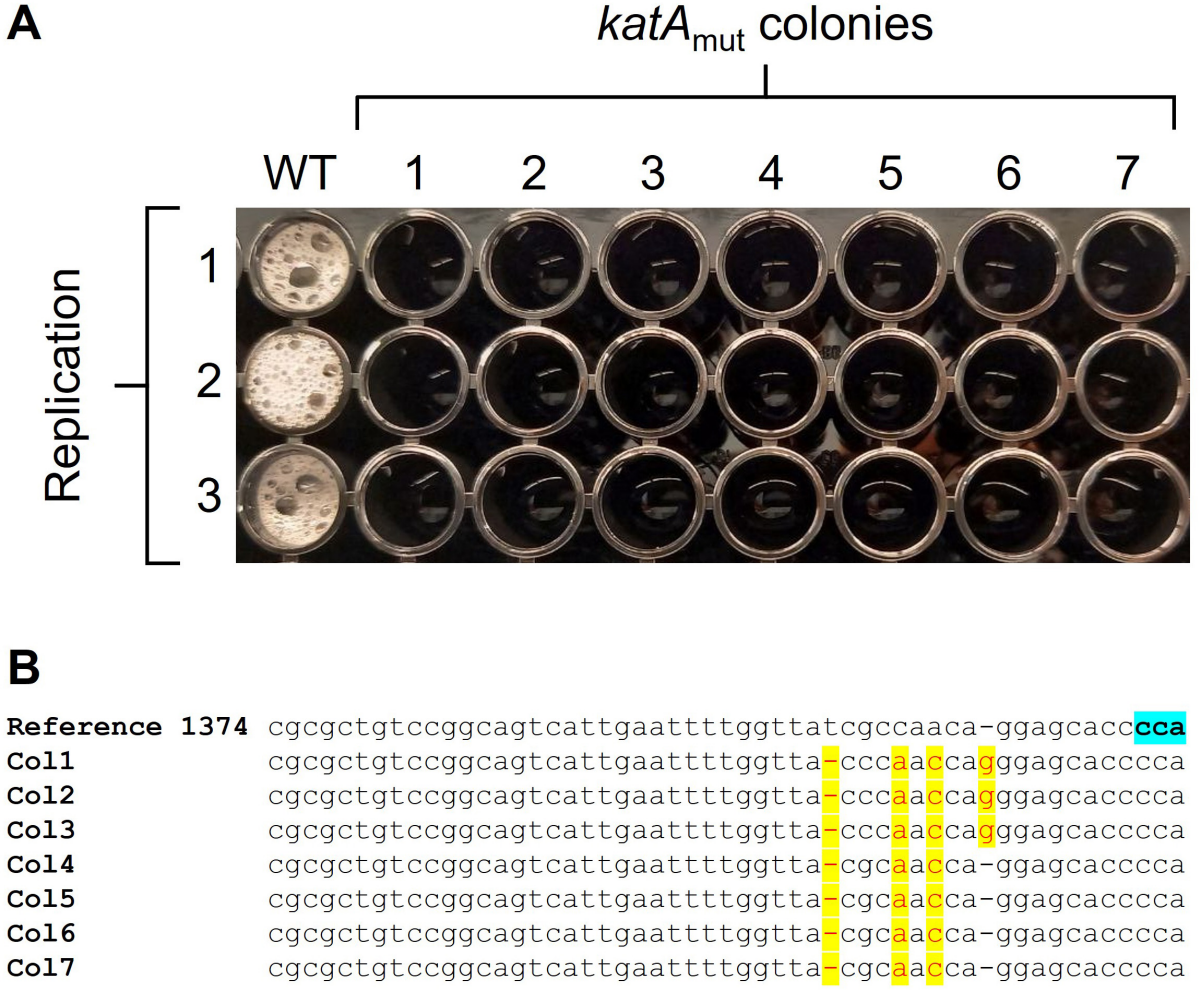
Assessing the effect of Cas9-mediated mutations in seven colonies from the *B. methanolicus katA* mutant strain (*katA*_mut_) in comparison to the control *B. methanolicus* strain MGA3 (WT). **(A)** H_2_O_2_ drop assay assessing catalase activity, the release of oxygen bubbles indicates catalase activity. **(B)** Partial alignment of Sanger sequencing results for the *katA* gene (nucleotides 1,374–1,429, reverse strand). The Cas9-mediated mutations are derived from the absence of a repair template in the plasmid system. Sequencing reads are aligned to the WT reference sequence. The PAM sequence is highlighted in blue in the reference, and sequencing mismatches are highlighted in yellow.

To expand the functionality of the CRISPR-Cas9 system in *B. methanolicus*, we employed it for gene deactivation by leveraging the bacterium's native DNA repair pathways, enabling error-prone end-joining in the absence of a repair template. The *B. methanolicus* chromosome encodes three main mechanisms for repairing DNA double-strand breaks (DSB). The first pathway is mediated by RecA (BMMGA3_06490), a multifunctional protein that mediates homologous recombination. Moreover, the genome search suggests that *B. methanolicus* may possess an additional template-dependent repair pathway involving the *addA* and *addB* gene cluster (BMMGA3_04250–04255), which encodes a RecBCD-like helicase/nuclease complex (Chayot et al., 2010). The third pathway, putatively active when no repair template is available, is the classical non-homologous end joining (NHEJ). It involves the genes *ykoV* (BMMGA3_09830) encoding DNA-end-binding protein Ku and *ykoU* (BMMGA3_09875) encoding DNA repair polymerase/ligase LigD (Hernández-Tamayo et al., 2022). We did not identify annotated genes encoding a complete microhomology-mediated end-joining pathway (Sfeir and Symington, 2015). To investigate the functionality of an error-prone end-joining mechanism in *B. methanolicus*, we chose the *katA* gene encoding a catalase (BMMGA3_04865) as a target and a pCasPP-based plasmid carrying only the *katA* targeting sgRNA without providing homologous flanks for HDR. Approximately 600 colonies were obtained, showing a much higher number than expected for typical escapers (Li et al., 2023). From those, 7 clones picked and were tested for catalase activity, and a function loss was observed for all of them in comparison to the WT control (Figure 4A). Sequencing of the *katA* gene PCR-amplicons from these clones revealed that the activity loss was possibly caused by mutations located in 9 to 18 bp downstream the targeted PAM region (Figure 4B). By that approach, the functionality of an error-prone DNA repair system in *B. methanolicus* was confirmed. All 7 colonies analyzed contained mutations at the Cas9 cleavage site, highlighting the potential of this system for rapid gene inactivation and future applications in multiplex genome editing.

**Figure 5:**
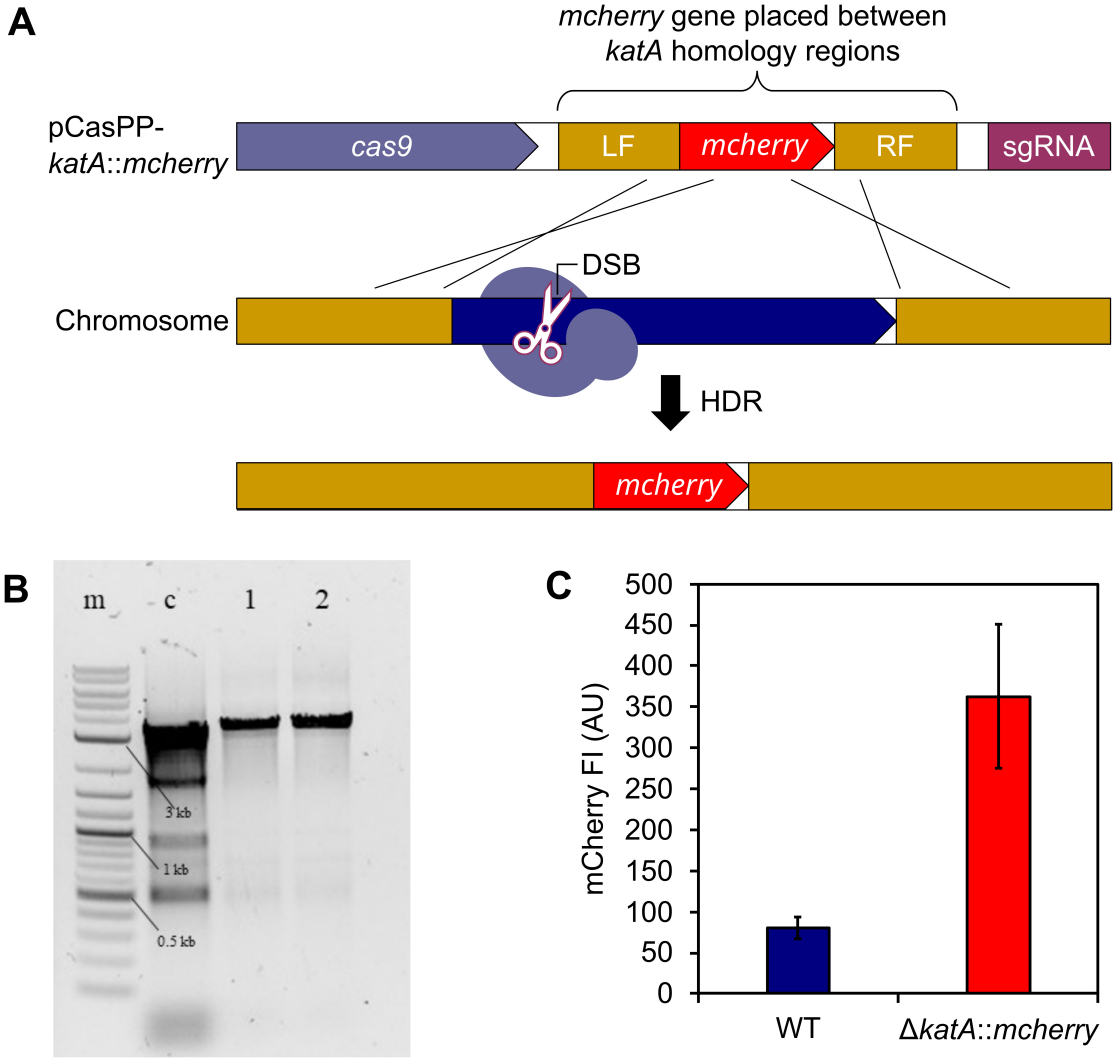
**(A)** Plasmid design for gene insertion in the genome of *B. methanolicus*. The HDR system was used to integrate the reporter gene *mcherry*, which is flanked by sequences homologous to the *katA* locus, enabling precise genomic insertion. The *mdh* promoter was placed upstream the *mcherry* gene. **(B)** CRISPR-Cas9-mediated *mcherry* insertion was confirmed by PCR screening of genomic regions flanking the homologous arms. WT (lane c) strains produced ~3.5 kb DNA fragments, whereas *mcherry* insertion clones yielded ~3.8 kb fragments. **(C)** mCherry fluorescence intensity (FI, in arbitrary units, AU) measured in WT and Δ*katA*::*mcherry* strains. The AU values (y-axis) were divided by 1,000.

The *katA* gene was also employed as a proof-of-concept target to demonstrate the applicability of the CRISPR-Cas system for gene insertions in *B. methanolicus*. In this experiment, the *katA* gene was replaced with the reporter gene *mcherry*. For this purpose, the construct pCasPP-Δ*katA::mcherry* contained the same *katA* homology arms and the sgRNA sequence used for *katA* deletion in the pCasPP-Δ*katA* plasmid, but with the homology arms flanking the P*mdh*-*mcherry* cassette (Table 1; Figure 5A). Successful gene insertion was verified by PCR. PCR primers bind to the chromosomal regions flanking the *katA* locus, outside the homology arms (Supplementary Table 1). The ~0.3 kb difference between WT (~3.5 kb) and *mcherry* insertion clones (~3.8 kb) corresponds to the replacement of the *katA* coding sequence with the *mcherry* ORF plus the *mdh* promoter, consistent with the expected fragment size (Figure 5B). The insertion frequency was comparable to that observed for deletion experiments, with approximately 3 non-recombinant clones among 20 colonies screened. The resulting strain, Δ*katA::mcherry*, exhibited approximately threefold higher mCherry fluorescence compared to the WT (Figure 5C). The background mCherry fluorescence observed in the WT had also been demonstrated previously (Irla et al., 2016). Hence, these results confirm the successful application of the CRISPR-Cas system for targeted gene insertion in *B. methanolicus*.

Next, we extended the CRISPR system to an additional genetic target. We targeted the gene encoding the major regulator Spo0A (BMMGA3_11365; WP_004435551.1), which is known to directly or indirectly influence more than 500 genes (Molle et al., 2003) and can be used in various microbial strains to decrease sporulation (Hou et al., 2016; Huang et al., 2004). Firstly, we have evaluated the efficiency of the CRISPR-Cas9 for the gene deletions (Supplementary Figure 3A). The plasmid pCasPP-*spo0A* was transferred to *B. methanolicus* via conjugation, and the empty pCasPP plasmid (no targeting sgRNA and no homologous flanks) was used as a control. Colony PCR analysis revealed 100% conjugation efficiency for the non-targeting pCasPP empty control (8/8 tested clones positive from 288 in total) as well as 100% editing efficiency for the targeting pCasPP-*spo0A* construct (10/10 tested clones positive from 28 in total). To evaluate the efficiency of the CRISPR-Cas9 system for targeted gene replacements, as previously described for *P. polymyxa* (Meliawati et al., 2022b), the pCasPP-*spo0A* construct was modified by inserting an mRFP1 encoding gene in between the homology flanks, thereby yielding the insertion plasmid pCasPP-*spo0A::mrfp1*. This plasmid carried the same sgRNA as utilized for the deletion of the *spo0A* gene but was designed to replace the *spo0A* gene with the mRFP1 encoding gene at its native locus. For that approach, a slightly lower editing efficiency of 95.2% was observed (20/21 positive clones from 2,784 in total). The positive integration of the *mrfp1* gene was confirmed via colony PCR and subsequent sequencing of the resulting amplicons (Supplementary Figure 3B). By that, the editing efficiencies were proven to be close to 100% for single gene deletions as well as for gene replacements in *B. methanolicus*.

## 4 Discussion

Here, we have developed and validated a strategy for scarless gene deletion in thermophilic *B. methanolicus* using a Cas9 protein. Even though binding of dCas9 to intact DNA occurs at temperatures above 40 °C, leading to successful downregulation of gene expression in *B. methanolicus*, the nucleolytic activity of Cas9 halts at temperatures above that threshold (Schultenkämper et al., 2019; Mougiakos et al., 2017b). We employed the wide temperature growth range of *B. methanolicus* to facilitate the CRISPR-Cas9-diven genome modifications. This strategy encompassed the co-cultivation at 40 °C during the conjugation step with *E. coli* S17.1 λ*pir* concurrent with functional expression of the Cas9 protein and introduction of DSB, followed by transfer to 50 °C to deactivate Cas9 protein, initiate DNA repair, and eliminate *E. coli*. An opposite strategy of temperature control of Cas9 activity was used for thermophilic *Bacillus smithii*, where genome modification was achieved via homologous recombination with plasmid-borne homology regions conducted at 45–55 °C, when Cas9 is not active. The Cas9 protein was consecutively used as a counterselection system activated through transfer to 37 °C. Then, the active Cas9 catalyzed the introduction of lethal DSB breaks to the WT cells (Mougiakos et al., 2017a). This shows that the limited thermal activity of Cas9, combined with a broad cultivation range of the host organism, can be leveraged to decrease the toxicity of Cas9 (Zhang and Voigt, 2018).

The functionality of the CRISPR-Cas9 system in *B. methanolicus* was expanded beyond simple gene deletions to gene insertions. The insertion of the *mcherry* gene at the *katA* locus exhibited an efficiency of approximately 85%, which was comparable to the efficiency achieved for *katA* deletion. Moreover, the gene integration of *mrfp1* at the *spo0A* locus has demonstrated the suitability of the pCasPP plasmid for CRISPR-Cas9-based gene integrations in *B. methanolicus* at a high efficiency of 95.2% compared to 100% of the gene deletion approach showing the great potential of this system for the strain construction. Our editing efficiencies are consistent with previously reported results for Cas9-mediated genome editing in bacteria, where single-gene deletions typically reach efficiencies close to 100%, and gene insertions achieve around 70–80% efficiency (Zou et al., 2022; Li et al., 2018). These comparable outcomes indicate that the CRISPR system implemented here performs at levels typical of previously established bacterial genome editing platforms.

In bacteria lacking a functional NHEJ pathway, Cas9-generated DSB typically result in cell death (Bowater and Doherty, 2006). This feature can be exploited as an effective counter-selection tool to eliminate unedited cells and enhance genome editing efficiency when HDR is applied (Cui and Bikard, 2016). However, in the absence of a repair template, all independently isolated clones in this study exhibited highly similar small indels immediately adjacent to the Cas9 cut site, a pattern characteristic of Cas9-triggered end-joining rather than spontaneous mutation (Figure 4B). Hence, using the CRISPR-Cas9 system, we have demonstrated that *B. methanolicus* possesses an active error-prone end-joining repair pathway. These results highlight an additional layer of genome plasticity in *B. methanolicus*, which can be exploited for rapid gene inactivation without relying on HDR. Although our genome analysis suggests the presence of an active NHEJ repair system, future studies should generate *ykoV* and *ykoU* mutants to determine whether the observed mutagenesis events depend on these genes.

The functionality of a newly established CRISPR-Cas9-mediated gene deletion system for *B. methanolicus* was confirmed through the targeted deletion of genes *katA* and *ald* and further physiological investigation. For all targeted genes, a high editing efficiency was observed. For increased flexibility of the genetic tool, the gene replacement approach of *katA* with *mcherry* and of *spo0A* with *mrfp1* was performed and showed efficiencies over 80%. The sequencing of the genomes of the deletion strains Δ*katA* and Δ*ald* revealed the presence of 13 and 14 variants, respectively. The genomic mutation rate of bacteria can be roughly estimated at around 0.003 per DNA replication, suggesting that the accumulation of 10^−6^ mutations/genome in the deletion strains is likely caused by the general evolutionary forces (Drake, 1991; Lynch et al., 2016). The gene deletions possibly exert selective pressure that favors the emergence of secondary mutations, which could represent adaptive responses inherent to the specific deletions. For example, we suggest that the mutation found in the genes *gapN* and *asnO* may be associated with the unmitigated oxidative stress observed in the *katA* deletion strain, while the mutated lysine biosynthesis gene *dapG* could be linked to the *ald* deletion strain. However, these potential relationships require further investigation to be confirmed.

Nevertheless, these deletions allowed us to confirm the physiological roles of both enzymes. A previous study has demonstrated that targeting *katA* with CRISPRi reduces specific catalase activity and suppresses oxygen formation from H_2_O_2_ in *B. methanolicus* (Schultenkämper et al., 2019). Catalases play a crucial role in defending against oxidative stress in bacteria by catalyzing the decomposition of reactive oxygen species (ROS), specifically H_2_O_2_. This reaction converts H_2_O_2_ into oxygen (O_2_) and water (H_2_O), mitigating the harmful effects of ROS. The deletion strain Δ*katA* showed moderate tolerance to 0.1 mg/L mM H_2_O_2_ supplemented to the growth media (Figure 3). This prompted a search for alternative enzymes that could potentially replace catalase activity in *B. methanolicus* (see Section 3.2). Genes annotated as peroxidases might contribute to the reduction of hydrogen peroxide and other ROS in the absence of catalase activity (Pedre et al., 2015; Yu et al., 2025). Further functional characterization of these enzymes will be crucial to understanding their roles in ROS detoxification and their potential to replace or complement catalase activity in *B. methanolicus*.

Interestingly, the approximately 42% growth rate reduction in the Δ*katA* strain in comparison to the WT, observed without supplementation of H_2_O_2_, indicates that catalase deletion affects cellular physiology beyond oxidative stress tolerance (Figure 3A). For instance, KatA functions as a major vegetative catalase that removes endogenously generated H_2_O_2_ and is crucial for oxidative stress resistance during aerobic growth and stationary phase of *Bacillus subtilis*. KatA is also secreted extracellularly, contributing to protection against oxidative damage in stationary phase and rich media (Naclerio et al., 1995). This phenotype observed in this study is consistent with a scenario in which loss of KatA leads to elevated steady-state H_2_O_2_ levels and chronic low-level oxidative stress, resulting in DNA, lipid and protein damage, and inactivation of iron-sulfur cluster-containing enzymes that are key for central metabolism and respiration (Mols and Abee, 2011; Dowds, 1994; Engelmann and Hecker, 1996). Such global effects on macromolecular integrity and redox-sensitive pathways likely underlie the slowed basal growth of the Δ*katA* strain, although the specific molecular targets remain to be identified. From a metabolic engineering perspective, these findings highlight that using *katA* as a handle to tune intracellular peroxide levels or stress responses may impose a significant fitness cost, so any potential benefits for product formation must be balanced against reduced growth and robustness.

The WT strain of *B. methanolicus* has been shown to secrete up to 0.14 M alanine in methanol fed batch fermentations, and similarly to other *Bacillus* species, alanine biosynthesis was thought to be the result of alanine dehydrogenase (Ald) activity through reductive amination of pyruvate in the methanol metabolism pathway (Brautaset et al., 2007; Ohashima and Soda, 1979; Dave and Kadeppagari, 2019) (Supplementary Figure 2A). In this study, repression of *ald* led to a reduced level of secreted alanine, further supporting the involvement of Ald in alanine biosynthesis in this organism (Supplementary Figure 1B). Although deletion of *ald* did not reproduce this trend (Supplementary Figure 2B), it resulted in decreased reductive amination of pyruvate to alanine (Table 2). The discrepancy in alanine production resulting from *ald* repression and deletion likely reflects differences in cultivation formats (standard shake flask cultivation for CRISPRi experiments vs. the Duetz plate system for the deletion strain), which impose distinct metabolic and aeration conditions. In addition, metabolic redundancy at the pyruvate node may buffer the effect of *ald* deletion. For example, recent work has found that AlaT (YugH) is the major amino transferase responsible for alanine biosynthesis in *B. subtilis*, and to a lesser degree a second aminotransferase Dat (YheM) (Sidiq et al., 2021). The knockout of *alaT* led to partial alanine auxotrophy and the activity of Dat was able to restore some growth in the absence of alanine, which otherwise seemed to inhibit its activity. This could indicate the existence of similar pathways in *B. methanolicus* enabling the production of alanine in an Ald knockout strain, which would be concurrent with our results (Figure 4). In *Bacillus licheniformis*, activation of a previously dormant aminotransferase pathway (Dat1) restored alanine synthesis in deletion strains, suggesting that similar compensatory mechanisms may exist (Han et al., 2023). Moreover, two alanine dehydrogenase paralogs in *B. licheniformis* were shown to be functionally interchangeable, further supporting the idea of redundancy (Kostner et al., 2017). Those studies suggest interesting targets for the future work that is required to further characterize the function of Ald and alanine metabolism in *B. methanolicus*, and we believe that the CRISPR-Cas9 tools we describe in this study will be instrumental in this work.

Several studies have demonstrated the effects of null mutation insertions and deletion of the *ald* gene in both the carbon and nitrogen metabolism of various organisms, however the role of Ald, and whether it is responsible for the production of alanine in *B. methanolicus* is yet to be fully elucidated. In *B. subtilis*, alanine dehydrogenase was first identified as a catalyser of the reversible conversion of alanine to pyruvate. The degradation of alanine through an oxidative deamination to provide pyruvate provided the cell with an important energy source for sporulation (Wiame and Piérard, 1955; Siranosian et al., 1993). This was demonstrated by inserting null mutations in the *ald* gene of *B. subtilis*, resulting in a sporulation defect (Wiame and Piérard, 1955; Siranosian et al., 1993). Studies in *M. tuberculosis* and *M. smegmatis* demonstrated that Ald enabled survival when transitioning from aerobic to anaerobic conditions, suggesting that Ald may also function to maintain an optimal cofactor balance during dormancy in preparation for regrowth (Hutter and Dick, 1998; Giffin et al., 2016). Additionally, ketone compounds such as acetoin are produced in *B. methanolicus* through the overexpression of the pyruvate-derived pathway, comprising the genes *alsS* (acetolactate synthase) and *alsD* (acetolactate decarboxylase) (Drejer et al., 2020). The deletion of *ald* may lead to the accumulation of pyruvate, thereby providing overflow of substrate for *alsS* activity and potentially increasing acetoin production. These insights highlight the potential of the *B. methanolicus* deletion system established in this study for metabolic engineering toward the production of methanol-based value-added chemicals.

## 5 Conclusions

The construction of the pCasPP-based plasmids enabled highly efficient genome editing in *B. methanolicus*, utilizing Cas9-mediated cleavage to target the *katA* and *ald* genes. Improved transformation efficiency through conjugation, as opposed to conventional electroporation, enabled successful implementation of CRISPR-Cas9 technology in this organism. Genome sequencing confirmed the successful deletion of *katA* and *ald*, validating the reliability of the system for gene manipulation in *B. methanolicus*. The gene deletion and complementation performed in this study revealed the crucial role of *katA* in oxidative stress tolerance in *B. methanolicus*. The results from this study also demonstrate that Ald in *B. methanolicus* primarily catalyzes the reductive amination of pyruvate to alanine. Furthermore, an efficient gene replacement of the *katA* gene with *mcherry* was realized with nearly 85% efficiency. The CRISPR-Cas9-based deletion and insertion approach developed in this study was successfully extended to another target gene, *spo0A*, demonstrating similarly high efficiencies for both insertion and deletion. Additionally, we have proven that *B. methanolicus* possesses a functional, error-prone end-joining mechanism that generates effective mutagenesis following DSB, resulting in loss-of-function phenotype. Our findings contribute to a better understanding of the metabolic pathways in *B. methanolicus* and highlight the potential of CRISPR-Cas9-based tools for functional genomic studies as well as metabolic engineering in this organism.

## Data Availability

The data presented in this study are publicly available. The data can be found here: https://www.ncbi.nlm.nih.gov/, accession PRJNA1268771.
