# Supplementary material for: CRISPR-Cas9-driven genome editing in Bacillus methanolicus MGA3
Source: Front Microbiol. 2026 Jan 28;16:1728984. doi: 10.3389/fmicb.2025.1728984 (PMC12893347; doi:10.3389/fmicb.2025.1728984)
Supplement: Supplementary file 1 [file Data_Sheet_1.pdf]

SUPPLEMENTARY MATERIAL

CRISPR-Cas9-driven genome editing in *Bacillus methanolicus* MGA3

May L. K. Khider[1], Marta Irla[2], Marina Gil López[1], Anna Gispert[1], Thomas Konjetzko[3], Meliawati Meliawati[3], Jochen Schmid[1,3], Trygve Brautaset[1] & Luciana Fernandes Brito[1]*

[1]Department of Biotechnology and Food Science, NTNU: Norwegian University of Science and Technology, Sem Sælandsvei 6/8, 7491 Trondheim, Norway

[2]Department of Biological and Chemical Engineering, Aarhus University, Gustav Wieds Vej 10 D, 8000 Aarhus C

[3]Institute of Molecular Microbiology and Biotechnology, University of Münster, Corrensstrasse 3, 48149 Münster, Germany

*Correspondence:

Corresponding author

luciana.f.d.brito@ntnu.no

Supplementary Methods

To assess whether expression attenuation affects amino acid production, the strains MGA3(piCas-*ald*), i*ald*, and its empty vector counterpart MGA3(piCas), iCas, were cultivated in methanol-based medium in Duetz system well plates. Supernatants were collected after 24 hours (Klein et al., 2023; Brito et al., 2021). The supernatants were then analyzed using high-performance liquid chromatography (HPLC) with FMOC-Cl (fluorenylmethyloxycarbonyl chloride) derivatization, following the conditions outlined by Brito et al. (2021).

References

Brito LF, Irla M, Nærdal I, Le SB, Delépine B, Heux S, Brautaset T (2021). Evaluation of heterologous biosynthetic pathways for methanol-based 5-aminovalerate production by thermophilic Bacillus methanolicus. Front Bioeng Biotechnol 9:686319. doi: 10.3389/fbioe.2021.686319

Klein VJ, Brito LF, Perez-Garcia F, Brautaset T, Irla M (2023). Metabolic engineering of thermophilic Bacillus methanolicus for riboflavin overproduction from methanol. Microb Biotechnol 16(5):1011-1026. doi: 10.1111/1751-7915.14239

Supplementary Tables

Table S1. Primers used in this study

Name	Sequence (5'-3')	Description
pcasppsgfw	gaattcagatctacgcgttccccgcaaaagcggcc	Sequencing primer for sgRNA region in the plasmid pCasPP
colHRfw	tgttacgccggcggtagccggccag	Sequencing/Forward colony PCR primer binding upstream the *Spe*I restriction region in the pCasPP plasmid
colHRrv	cacacagcccagcttggagcgaacg	Sequencing/Reverse colony PCR primer binding downstream the *Spe*I restriction region in the pCasPP plasmid
katAsgfw	aataagaaaaaacttactacaagcgttttagagctagaaatagcaag	Forward primer for pCasPP plasmid PCR overlap extension containing *katA*-sgRNA sequence
katAsgrv	gcttgtagtaagttttttcttattcgcaatccccttt cagatactcgcac	Reverse primer for pCasPP plasmid PCR overlap extension containing *katA*-sgRNA sequence
LFkatAfw	accggcgcatcaagcccgccgagccagctatcatgaaaagacaatgatcgatgtctg	Forward primer for amplification of 1 kb left flank (homologous arm) for *katA* deletion; overlaps with *Spe*I restriction region in the pCasPP plasmid
LFkatArv	ttgggggtcttgccctcatctttaatgatg	Reverse primer for amplification of 1 kb left flank (homologous arm) for *katA* deletion; overlaps with SpeI restriction region in the pCasPP plasmid
RFkatAfw	gattagatcacctcagataaattgattattg	Forward primer for amplification of 1 kb right flank (homologous arm) for *katA* deletion; overlaps with SpeI restriction region in the pCasPP plasmid
RFkatArv	ctttttacggttcctggccagggagtcaaaccagcgatccattatatt	Reverse primer for amplification of 1 kb right flank (homologous arm) for *katA* deletion; overlaps with SpeI restriction region in the pCasPP plasmid
mdhPfw	caataatcaatttatctgaggtgatctaatcgttcattaaagagcagctgatgatgac	Forward primer for amplification of the P*mdh-mcherry* construct; overlaps with the *katA* gene left flank
mcherryrv	catcattaaagatgagggcaagacccccaattaagcaccggtggagtgacgaccttcagc	Reverse primer for amplification of the *mcherry* gene; overlaps with the *katA* gene right flank
katAdelfw	aacggaagcgttaacggaggc	Forward colony PCR primer for seletion of *katA* deletion clones
katAdelrv	gttcccagtgggagtcaaacc	Reverse colony PCR primer for seletion of *katA* deletion clones
katAseqfw	ggccctttgtatggaactgc	Forward sequencing primer binding outside of flanks for PCR confirmation of *katA* deletion
katAseqrv	agccggatatggaacacg	Reverse sequencing primer binding outside of flanks for PCR confirmation of *katA* deletion
ikatAfw	gggatataaacgttttatgataaatatacttttgctctctgcacacc	Forward primer for piCas plasmid PCR overlap extension containing *katA*-sgRNA sequence
ikatArv	taacttgctatttctagctctaaaacggtgtgcagagagcaaaagt	Reverse primer for piCas plasmid PCR overlap extension containing *katA*-sgRNA sequence
ikatAseq	gaggtgaccccaagaagaagaggaaggtgggctg	Sequencing primer binding upstream the sgRNA region in the piCas plasmid
aldsgfw	gattgtagacgtacgcggaaaattcttagtgcgagtatctgaaaggggatacgc	Forward primer for plasmid PCR overlap extension containing *ald*-sgRNA sequence
aldsgrv	ttccgcgtacgtctacaatcttattttaacttgctatttctagctctaaaac	Reverse primer for plasmid PCR overlap extension containing *ald*-sgRNA sequence
LFaldfw	cgcaaccggcgcatcaagcccgccgagatctcggatattacgcgaacagtagcttatggc	Forward primer for amplification of 1 kb left flank (homologous arm) for *ald* deletion; overlaps with SpeI restriction region in the pCasPP plasmid
LFaldrv	atgcgaattggagtaccttgaacaaatgtaaagaacaaagaggctg	Reverse primer for amplification of 1 kb left flank (homologous arm) for *ald* deletion; overlaps with SpeI restriction region in the pCasPP plasmid
RFaldfw	ttacatttgttcaaggtactccaattcgcatatttaaaacctccc	Forward primer for amplification of 1 kb right flank (homologous arm) for *ald* deletion; overlaps with SpeI restriction region in the pCasPP plasmid
Rfaldrv	cgcggcctttttacggttcctggccactagttctggatataaaactctcatacacattac	Reverse primer for amplification of 1 kb right flank (homologous arm) for *ald* deletion; overlaps with SpeI restriction region in the pCasPP plasmid
alddelfw	attatactacaatagaaatgcaattccttc	Forward colony PCR primer for seletion of *ald* deletion clones
alddelrv	agaccagtaatggaaaatatcttggaactg	Reverse colony PCR primer for seletion of *ald* deletion clones
aldseqfw	cgaaatacccgaaagagcttc	Forward sequencing primer binding outside of flanks for PCR confirmation of *ald* deletion
aldseqrv	cataacgggtgatatgctctgag	Reverse sequencing primer binding outside of flanks for PCR confirmation of *ald* deletion
katAfw	gtaaacaattacataaataggaggtagtagtacatgaccacaaataagaaaaaacttactacaagc	Forward primer for amplification of the *katA* gene; ovrelaps with the *BamH*I region of the pTH1mp plasmid
katArv	ggatccccgggaattcaagctttaaacatgttaaactttcttttgtacaggtaaacctagac	Reverse primer for amplification of the *katA* gene; ovrelaps with the *BamH*I region of the pTH1mp plasmid
aldfw	gtaaacaattacataaataggaggtagtagtacatgcgaattggagtacctgcagaattaaag	Forward primer for amplification of the *ald* gene; ovrelaps with the *BamH*I region of the pTH1mp plasmid

aldrv	ggatccccgggaattcaagctttaaacatgttac atttgttcaagaagtgtttttgtatc	Reverse primer for amplification of the *ald* gene; ovrelaps with the *BamH*I region of the pTH1mp plasmid
spo0AUSfw	gcgcatcaagcccgccgactacagaaaagattc cagaggcagc	Forward primer for amplification of 1 kb left flank (homologous arm) for *spo0A* deletion; overlaps with pCasPPF2rv primer
pCasPPF2rv	agtcggcgggcttgatg	General primer amplifying a fragment of pCasPP, used for insertion of 1 kb left flanks (homologous arms)
spo0ADSfw	caaatgatgaaactattcccctccccgatccatta atggtctc	Forward primer for amplification of 1 kb right flank (homologous arm) for *spo0A* deletion overlaps with spo0AUSrv
spo0AUSrv	gggaatagtttcatcatttgcatttgttgc	Reverse primer for amplification of 1 kb left flank (homologous arm) for *spo0A* deletion; overlaps with spo0ADSfw
pCasPPF3fw	agtggccaggaaccgtaaaaag	General primer amplifying a fragment of pCasPP, used for insertion of 1 kb right flanks (homologous arms)
spo0ADSrv	ctttttacggttcctggccactcaaagctgcagcc catgaaag	Reverse primer for amplification of 1 kb right flank (homologous arm) for *spo0A* deletion overlaps with pCasPPF3fw primer
mrfp1fw	atggcttcctccgaagacgttatc	Forward primer for *mrfp1* verification
spo0AUSisrv	gataacgtcttcggaggaagccataaggaatag tttcatcatttgcatttgttgc	Reverse primer for amplification of 1 kb left flank (homologous arm) for *spo0A* replacement by *mrfp1*; overlaps with mrfp1fw primer
spo0ADSisfw	cgtcactccaccggtgcttaatttttgtttcctcccc gatccattaatg	Forward primer for amplification of 1 kb right flank (homologous arm) for *spo0A* replacement by *mrfp1*; overlaps with mrfp1rv primer
spo0Averfw	gcaattcttagtcgggctgc	Forward colony PCR primer for seletion of *spo0A* deletion clones
spo0Aseqfw	gaatgggttcctttttggatgtcgtatg	Forward sequencing primer for verification of *spo0A* deletion clones
spo0Averrv	cgttgcgccgattgtcc	Reverse colony PCR primer for seletion of *spo0A* deletion clones
mrfp1rv	ttaagcaccggtggagtgacg	Reverse primer for *mrfp1* amplification
spo0Asgrv	cgctacttcgatcgcatgacgcgtatcccctttca gatactcgc	Reverse primer containing *spo0A*-sgRNA sequence, overlaps with spo0Asgfw primer
spo0Asgfw	gtcatgcgatcgaagtagcggttttagagctaga aatagcaagttaaaataaggctag	Forward primer containing *spo0A*-sgRNA sequence, overlaps with spo0Asgrv primer

Table S2. Nucleotide variants in the WT of *B. methanolicus*. POS – position of the single-nucleotide polymorphism in the WT genome; REF – nucleotide in the reference genome; ALT – nucleotide in the WT genome; QS – quality score.

POS	REF	ALT	QS	Gene ID	Function
432692	C	T	123	BMMGA3_02225	Hypothetical protein
432721	C	T	57	BMMGA3_02225	Hypothetical protein
432985	T	C	52	BMMGA3_02225	Hypothetical protein
432988	T	C	72	BMMGA3_02225	Hypothetical protein
432997	C	T	84	BMMGA3_02225	Hypothetical protein
433003	T	C	141	BMMGA3_02225	Hypothetical protein
433006	C	T	161	BMMGA3_02225	Hypothetical protein
433124	T	A	128	BMMGA3_02225	Hypothetical protein
433135	A	G	98	BMMGA3_02225	Hypothetical protein
453467	T	A	225	BMMGA3_02310	Spore cortex-lytic enzyme
453469	C	T	218	BMMGA3_02310	Spore cortex-lytic enzyme
453499	T	C	83	BMMGA3_02310	Spore cortex-lytic enzyme
453701	C	T	228	BMMGA3_02310	Spore cortex-lytic enzyme
1494460	T	C	64	BMMGA3_07525	Hypothetical protein
1494461	G	C	55	BMMGA3_07525	Hypothetical protein
1494462	A	G	57	BMMGA3_07525	Hypothetical protein
1494510	C	T	228	BMMGA3_07525	Hypothetical protein
1494522	T	C	181	BMMGA3_07525	Hypothetical protein
1494527	C	G	228	BMMGA3_07525	Hypothetical protein
1494544	A	G	169	BMMGA3_07525	Hypothetical protein
1494553	G	A	160	BMMGA3_07525	Hypothetical protein
1597000	GTT	GT	53	*yhxC*	Putative oxidoreductase YhxC
1605157	T	TC	69	BMMGA3_08075	Amino acid permease-associated region
1844338	C	G	122	BMMGA3_09225	Hypothetical protein
1844349	C	A	107	BMMGA3_09225	Hypothetical protein
1844361	T	A	166	BMMGA3_09225	Hypothetical protein
1844368	G	T	161	BMMGA3_09225	Hypothetical protein
1844369	C	T	206	BMMGA3_09225	Hypothetical protein
1844379	C	G	228	BMMGA3_09225	Hypothetical protein
1844406	C	T	153	BMMGA3_09225	Hypothetical protein
1844431	A	T	80	BMMGA3_09225	Hypothetical protein
1844951	A	G	228	BMMGA3_09230	Hypothetical protein
1845004	C	T	228	BMMGA3_09230	Hypothetical protein
1845040	T	C	228	BMMGA3_09230	Hypothetical protein
1845104	C	T	61	BMMGA3_09230	Hypothetical protein
1912572	ATC	ATCTC	59	BMMGA3_09570	Putative membrane protein
2051477	T	TATC	70	*mntR*	Transcriptional regulator MntR
2646584	T	C	54	*ezrA*	Septation ring formation regulator EzrA
3003059	A	G	148	*tagO*	Putative undecaprenyl-phosphate N-acetylglucosaminyl 1-phosphate transferase
3003245	G	A	55	*tagO*	Putative undecaprenyl-phosphate N-acetylglucosaminyl 1-phosphate transferase
3003285	T	C	214	*tagO*	Putative undecaprenyl-phosphate N-acetylglucosaminyl 1-phosphate transferase
3038207	C	CGATCT	70	BMMGA3_15270	Hypothetical protein

Supplementary Figures

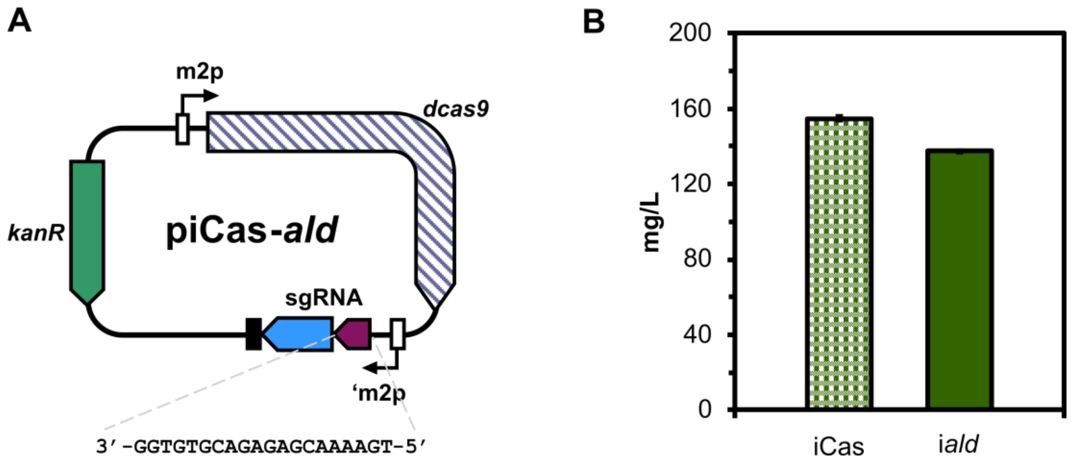

Figure S1. A- dCas9-mediated attenuation of *ald* gene expression with plasmid piCas-*ald*. B- Alanine titers (mg/L) in *B. methanolicus* strains iCas and i*ald* cultured in shake flasks. Bars represent the mean titers of technical triplicates, with error bars indicating the standard error. Significant differences were observed between strains, determined by the Scott-Knott test ($p < 0.01$).

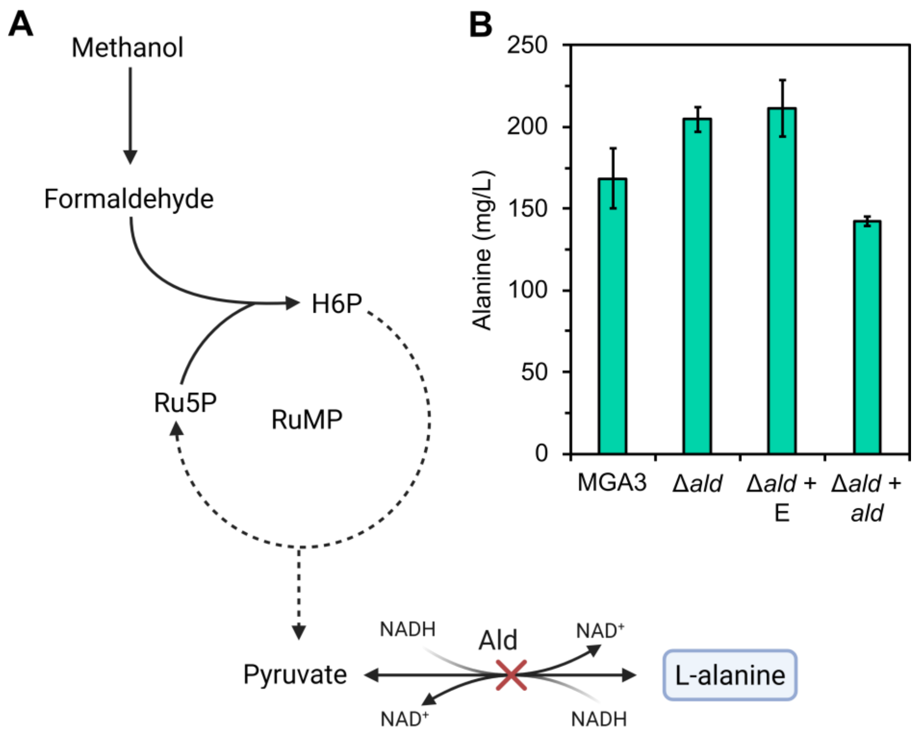

Figure S2. A- Metabolic pathways in *B. methanolicus* leading to the biosynthesis of alanine from methanol. Deletion of the *ald* gene could potentially impair alanine dehydrogenase activity, which catalyses the reversible reductive amination of pyruvate to alanine. Pathway scheme created with BioRender (https://BioRender.com/k80duxe). B- Alanine titers (mg/L) in *B. methanolicus* strains MGA3, Δ*ald,* Δ*ald* + E, Δ*ald* + *ald* cultured in Duetz plates. No significant differences were observed between strains for alanine titer, as determined by the Scott-Knott test ($p < 0.01$).

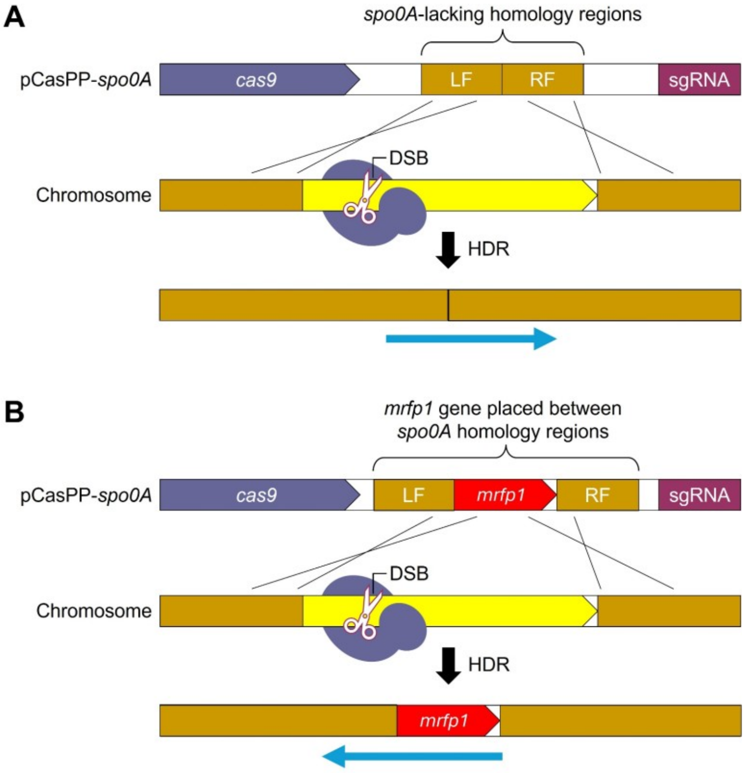

Figure S3. Plasmid design enabling different CRISPR-Cas9-mediated genome modifications in *B. methanolicus* MGA3. A- For scarless deletion of the *spo0A* gene, the DSB is repaired via the HDR pathway. A donor DNA template with flanking homologous sequences is provided on the plasmid, enabling precise replacement or deletion of the target region. B- The HDR system can also be used to insert the reporter gene *mrfp1*. The *mrfp1* gene is flanked by sequences homologous to the *spo0A* region, allowing for accurate integration into the genome. Blue arrows indicate Sanger sequencing coverage across the deletion or insertion regions, with lengths proportional to the coverage.
